# Supplementary material for: Focusing the electromagnetic field to 10−6λ for ultra-high enhancement of field-matter interaction
Source: Nat Commun. 2021 Nov 4;12:6389. doi: 10.1038/s41467-021-26662-5 (PMC8569218; doi:10.1038/s41467-021-26662-5)
Supplement: Supplementary file 1 — Supplementary Information [file 41467_2021_26662_MOESM1_ESM.pdf]

Supplementary Information for:

"Focusing the electromagnetic field to $10^{-6}\lambda$ for ultra-high enhancement of field-matter interaction"

Xiang-Dong Chen[1,2], En-Hui Wang[1,2], Long-Kun Shan[1,2], Ce Feng[1,2], Yu Zheng[1,2], Yang Dong[1,2], Guang-Can Guo[1,2], Fang-Wen Sun[1,2]

[1] *CAS Key Laboratory of Quantum Information, School of physics, University of Science and Technology of China, Hefei, 230026, People's Republic of China.*

[2] *CAS Center For Excellence in Quantum Information and Quantum Physics, University of Science and Technology of China, Hefei, 230026, People's Republic of China.*

Supplementary Note 1. THE SHAPE OF THE ANTENNA

The whole bowtie structure contains two parts: a small bowtie structure of Au film and a large bowtie structure of copper tap, as shown in Supplementary Fig. 1a. The gap of the small bowtie structure is W_{gap}=8 μm, and the width of the bowtie structure is W_1 =160 μm at the gap. The length of the whole structure L is changed by cutting the copper tape. To optimize the length of the bowtie structure for NV center spin manipulation, we record the Rabi oscillation frequency of the NV center with different bowtie lengths (without the Ag nanowire), as shown in Supplementary Fig. 1b and c. The maximum Rabi oscillation frequency is obtained with a length of 6.3 cm.

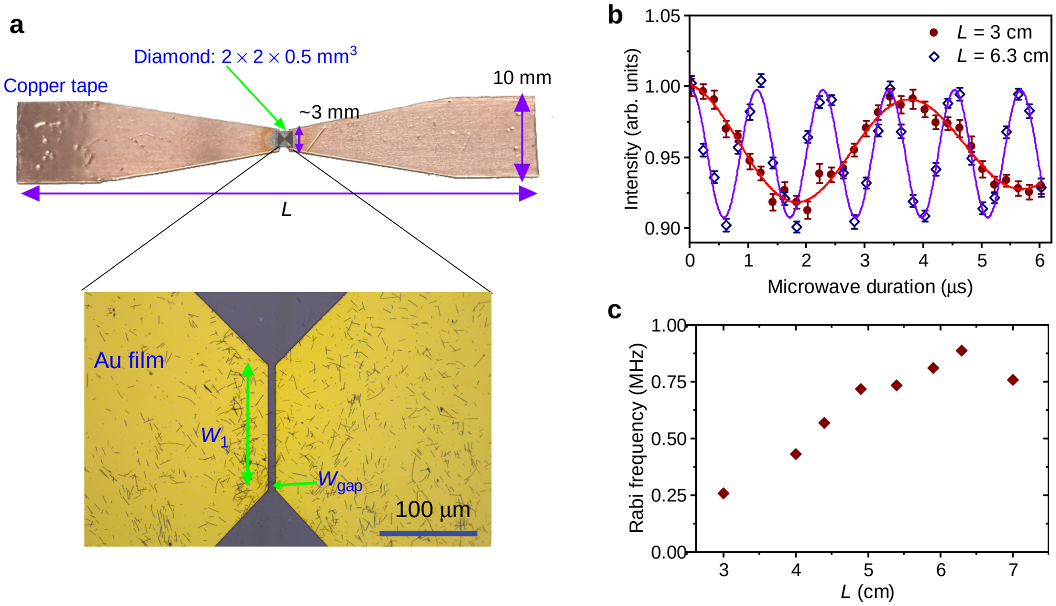

Supplementary Figure 1. Optimizing the nanowire-bowtie structure. a The shape of the nanowire-bowtie antenna. **b** Rabi oscillation of the NV center in the gap of bowtie structure with L =3 and 6.3 cm. The power of the free-space microwave is 21 W. Error bars represent the standard error. **c** The Rabi oscillation frequency changes with L.

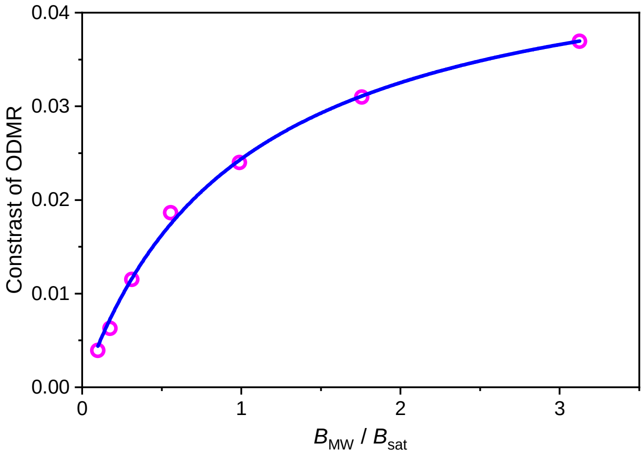

Supplementary Figure 2. **The contrast of ODMR signal with different microwave amplitudes.** The solid line is the fit of Supplementary Eq. (2).

Supplementary Note 2. THE MICROWAVE POWER DEPENDENT ODMR CONTRAST

The ODMR contrast of NV center is defined as

$$C = \frac{I_0 - I_{\rm MW}}{I_0}, \tag{1}$$

where $I_{\rm MW}$ and I_0 are the fluorescence intensity with and without resonant microwave pumping, respectively. To calibrate the power dependence of the OOMR contrast, we use a ring shape near-field antenna with a high spatial uniformity to pump the spin transition of NV center. The ODMR contrast of the NV center is recorded with different microwave amplitude $\rm B_{MW}$, as in Supplementary Fig. 2. The contrast is then fitted by a saturation function

$$C = C_0 \frac{B_{\rm MW}}{B_{\rm MW} + B_{\rm sat}}, \tag{2}$$

where C_0 is the saturated contrast and $B_{\rm sat}$ is the saturation microwave amplitude.

The results also indicate that, in the weak pumping region ($B_{\rm MW} \lesssim B_{\rm sat}$), the power dependent ODMR contrast can be simplified as a linear function:

$$C \approx \alpha B_{\rm MW}. \tag{3}$$

Then the fluorescence with microwave $B_{\rm MWi}$ is written as $I_{\rm MWi} \approx I_0 \cdot (1 - \alpha B_{\rm MWi})$. In Fig. 2e and f of the main text, we simultaneously record the fluorescence intensities with

two different microwaves, for example B_{MW1} and B_{MW2}. Then, the ratio of fluorescence intensities is approximated as

$$\frac{I_{\mathrm{MW1}}}{I_{\mathrm{MW2}}} = \frac{1-\alpha B_{\mathrm{MW1}}}{1-\alpha B_{\mathrm{MW2}}} \approx 1 + \alpha(B_{\mathrm{MW2}} - B_{\mathrm{MW1}}). \quad (4)$$

Here, the result is further simplified by considering $\alpha B_{\mathrm{MWi}} \ll 1$.

Supplementary Note 3. THE RESOLUTION OF CSD NANOSCOPY

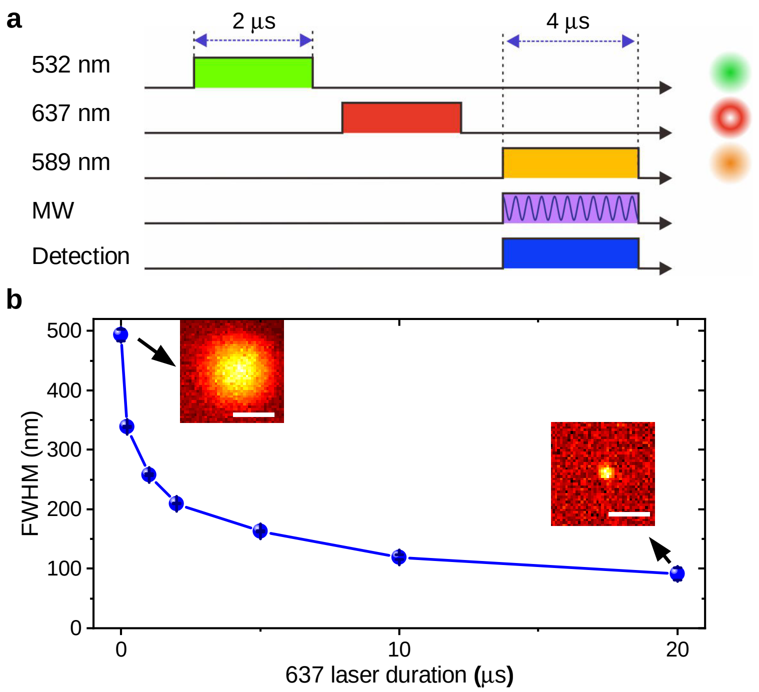

Supplementary Figure 3. The CSD nanoscopy for the diffraction-unlimited ODMR measurement. a The sequence for the CSD nanoscopy. **b** The CSD nanoscopy resolution changes with the 637 nm depletion laser duration. The power of the 637 nm depletion laser is 20 mW here. Error bars represent the standard error. The inserts show the images of a single NV center. The scale bars in the inserts are 400 nm in length.

The CSD nanoscopy is based on the optical manipulation of NV center charge state. Two charge states of NV center is utilized: the negatively charged NV^-, and the neutrally charged NV^0. The conversion between the two charge states can be pumped by laser pulse. And the two charge states can be optically distinguished according to the fluorescence intensity and

wavelength. In our experiments, a long pass optical filter is used to block the fluorescence of NV^0 charge state. Therefore, we treat the NV^0 as a dark state in the CSD nanoscopy.

The laser and microwave sequence for super-resolution imaging and sensing is shown in Supplementary Fig. 3a. A Gaussian-shaped green laser initializes NV center to the negatively charged NV^- state. Then a doughnut-shaped red laser changes the charge state to the neutral NV^0 charge state. The NV at the center of the doughnut-shaped laser beam remains to be NV^-. After that, the spin state transition of NV^- ground state is pumped by the local microwave field. The charge state and spin state of NV center is detected with a 589 nm laser.

In this work, the power of the 532 and 589 nm lasers are set to 0.2 and 0.05 mW, respectively. The spatial resolution of the CSD nanoscopy increases with the duration and power of the 637 nm doughnut-shaped depletion laser. Here, we set the power of the 637 nm laser to 20 mW. With a duration of 10 μs, the lateral resolution of NV imaging is improved to approximate 100 nm. It is high enough to map the distribution of the localized microwave field near the Ag nanowire.

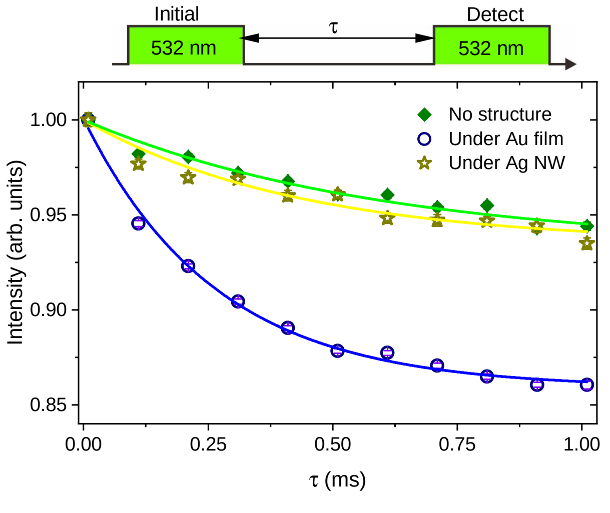

Supplementary Figure 4. **The spin relaxation of NV center.** The spin state of NV center is firstly initialized to $m_s = 0$ with a 532 nm laser pulse. After a decay time τ, the spin state is detected with another 532 nm laser pulse. The fluorescence intensity decreases as the spin state decays to a mixed state. Error bars represent the standard error.

Supplementary Note 4. THE JOHNSON NOISE

The random motion of electron in a conductor will cause electromagnetic fluctuations, known as Johnson noise. It decreases the spin relaxation time of the nearby NV center, and subsequently affects the applications with NV or other solid state spin. We measure the spin relaxation of NV centers at different positions with the nanowire-bowtie antenna, as shown in Supplementary Fig. 4. As expected, the Au film significantly decreases the relaxation time T_1 of NV center. In contrast, the spin relaxation time of NV center under the Ag nanowire does not show significant change. It suggests that the impact of Johnson noise from an Ag nanowire is small. The nanowire-bowtie antenna can be used for spin manipulation without reducing the relaxation time.

Supplementary Note 5. THE SIMULATION

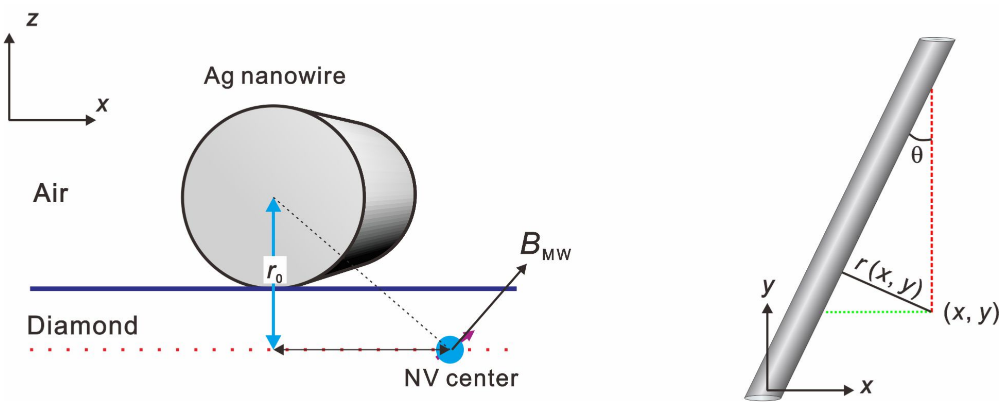

Supplementary Figure 5. **The illustration of the microwave that is produced by a straight line current.**

To simulate the magnetic component of the microwave field that is produced by a straight line current, we consider an Ag nanowire in the xy plane, as in Supplementary Fig. 5. The angle between the y axis and the nanowire is θ. The position of nanowire is written as (x_0, y_0), which follows $y_0 = \cot\theta \cdot x_0$. For a straight line current on the Ag nanowire, the magnetic amplitude of the microwave at position (x, y) is

$$B_{\mathrm{MW}}(x, y) \propto \frac{1}{\sqrt{r_0^2 + r^2}}, \qquad (5)$$

where $r = \sin\theta \cdot (\cot\theta \cdot x - y)$. r_0 is determined by the radius of the nanowire and the depth of NV center. The projections of magnetic vector can be written as:

$$B_{\mathrm{MW},x} = B_{\mathrm{MW}} \frac{r_0}{\sqrt{r_0^2 + r^2}} \cdot \cos\theta, \tag{6}$$

$$B_{\mathrm{MW},y} = B_{\mathrm{MW}} \frac{r_0}{\sqrt{r_0^2 + r^2}} \cdot \sin\theta, \tag{7}$$

$$B_{\mathrm{MW},z} = B_{\mathrm{MW}} \frac{r}{\sqrt{r_0^2 + r^2}}. \tag{8}$$

The amplitude of the microwave component that effectively pumps NVi center spin transition is then written as:

$$B_{\mathrm{MW1}} = \sqrt{B_{\mathrm{MW}}^2 - (-\sqrt{\frac{2}{3}} B_{\mathrm{MW},x} + \sqrt{\frac{1}{3}} B_{\mathrm{MW},z})^2}, \tag{9}$$

$$B_{\mathrm{MW2}} = \sqrt{B_{\mathrm{MW}}^2 - (\sqrt{\frac{2}{3}} B_{\mathrm{MW},x} + \sqrt{\frac{1}{3}} B_{\mathrm{MW},z})^2}, \tag{10}$$

$$B_{\mathrm{MW3}} = \sqrt{B_{\mathrm{MW}}^2 - (\sqrt{\frac{2}{3}} B_{\mathrm{MW},y} + \sqrt{\frac{1}{3}} B_{\mathrm{MW},z})^2}, \tag{11}$$

$$B_{\mathrm{MW4}} = \sqrt{B_{\mathrm{MW}}^2 - (-\sqrt{\frac{2}{3}} B_{\mathrm{MW},y} + \sqrt{\frac{1}{3}} B_{\mathrm{MW},z})^2}. \tag{12}$$

The fluorescence intensity distribution without microwave is written as $I_0(x, y)$. The fluorescence of NVi center under microwave pumping is:

$$I_{\mathrm{NVi}}(x, y) = I_0(x, y) \cdot (1 - C_{\mathrm{NVi}}), \tag{13}$$

where C_{NVi} is the ODMR contrast of NVi center. C_{NVi} is determined by the amplitude of B_{MWi}, as in Supplementary Eq. (2).

In our experiments, the CSD nanoscopy is applied for the microwave distribution measurement. The point spreading function of the CSD nanoscopy is written as

$$\mathrm{PSF}(x, y) = \frac{1}{\sqrt{2\pi}\sigma} e^{-\frac{x^2+y^2}{2\sigma^2}}. \tag{14}$$

The full width at half maximum (FWHM = 2.355 σ) presents the resolution of the CSD nanoscopy. The detected fluorescence signal is the convolution of the NV center fluorescence distribution and the point spreading function of the CSD nanoscopy:

$$I_{\mathrm{det,NVi}}(x, y) = \iint I_{\mathrm{NVi}}(x_1, y_1) \cdot \mathrm{PSF}(x - x_1, y - y_1) dx_1 dy_1. \tag{15}$$

The convolution of the fluorescence distribution without microwave pumping is also calculated as

$$I_{\mathrm{det},0}(x,y) = \iint I_0(x_1,y_1)\cdot \mathrm{PSF}(x-x_1,y-y_1)dx_1dy_1. \tag{16}$$

Then, the detected ODMR signal distribution is simulated as

$$C_{\mathrm{det},i}(x,y) = \frac{I_{\mathrm{det},0}(x,y) - I_{\mathrm{det,NVi}}(x,y)}{I_{\mathrm{det},0}(x,y)}. \tag{17}$$

In the main text, we show that the simulation of a straight line current matches well with the experimental results.
